# Investigation of the Pathobiology of *Edwardsiella piscicida*—Septicemia in Largemouth Bass

**DOI:** 10.3390/pathogens14040334

**Published:** 2025-03-31

**Authors:** Grace Ramena, Suja Aarattuthodi, Gnanender Sriramoju, Yathish Ramena

**Affiliations:** 1 Aquaculture/Fisheries Center, University of Arkansas Pine Bluff, Pine Bluff, AR 71601, USA; ramenag@uapb.edu (G.R.); s.gnanender@gmail.com (G.S.); ramenay@uapb.edu (Y.R.); 2 Plant Genetics Research Unit, United States Department of Agriculture—Agricultural Research Service, Columbia, MO 65211, USA

**Keywords:** *Edwardsiella piscicida*, virulence, largemouth bass, septicemia, aquaculture

## Abstract

*Edwardsiella piscicida* septicemia is a significant threat to aquaculture, causing substantial production and economic losses. The causative bacterium *E. piscicida* infects multiple fish species of aquaculture relevance. This study investigated the pathogenicity of catfish-derived *E. piscicida* in largemouth bass (*Micropterus salmoides*) fingerlings. The experimental infection of largemouth bass using genotypically distinct *E. piscicida* isolates resulted in significant fish mortality independent of the isolate genotypes. A specific correlation between discrete *E. piscicida* genotypes and fish mortalities was not identified. The histopathological assessment of tissues from infected fish revealed inflammatory lesions characteristic of bacterial septicemia. This study provides insights into the pathogenesis of heterologous *E. piscicida* isolates in largemouth bass fingerlings, which could be crucial in developing effective pathogen-targeted management strategies to combat a deadly disease.

## 1. Introduction

*Edwardsiella piscicida* is a virulent pathogen of significant concern in aquaculture operations. This Gram-negative, facultative anaerobic bacterium belongs to the family Hafniaceae, previously classified under Enterobacteriaceae [1,2]. It causes Edwardsiellosis (*E. piscicida* septicemia) in several freshwater and marine fish species worldwide, posing a major threat to global aquaculture [3,4,5,6,7,8,9,10,11,12,13,14,15,16,17,18,19].

The bacterium enters a susceptible host through body surface wounds or the gastrointestinal tract, leading to systemic infection [5,9,20,21]. Clinical signs associated with *E. piscicida* septicemia in fish include exophthalmia, hemorrhages, skin ulcers, discoloration of the body surface, abdominal distension, erratic swimming, loss of appetite, and, in severe cases, the characteristic ‘hole-in-the-head’ lesion [5,9,17].

The wide host range and geographical distribution of *E. piscicida* pose it as one of the most economically devastating pathogens affecting multiple commercially important fish species [4,7,8,9,10,11,14,17,18,22]. With the intensification of aquaculture practices, the resulting economic losses due to diseases can be substantial. Preventative measures to manage the risk of *E. piscicida* in aquaculture typically include a combination of biosecurity practices, vaccines, antimicrobial agents, probiotics, and maintenance of good water quality. Although some effective antibiotics are available, regulatory restrictions associated with antimicrobial resistance and the cost-prohibitive nature of medicated feed make those infeasible for disease management [23].

Understanding the pathogenicity of genetically distinct *E. piscicida* strains and their virulence factors in commercially important fish species is crucial for several aquaculture sectors [3,4,5,6,7,8,9,10,11,14,15,16,17,18,19]. Studies have looked into these aspects to facilitate the development of effective and economically pragmatic pathogen-targeted management strategies to limit the impacts of *E. piscicida* in aquaculture [24,25,26]. Largemouth bass (*Micropterus salmoides*) is an important food and sportfish species in the U.S. With over 190 farms spread over 32 states, the industry generates ~USD 35 million annually [27]. There is limited understanding of the pathogenicity of distinct *E. piscicida* strains in largemouth bass, which is particularly important considering the potential impact of this pathogen on bass production.

This study examined the virulence of catfish-originated *E. piscicida* isolates from different genetic clades in largemouth bass fingerlings via experimental infection studies. Systematic investigation of the pathobiology of *E. piscicida* septicemia in largemouth bass can contribute to our understanding of this important fish disease and help mitigate its impact on aquaculture.

## 2. Methods

### 2.1. Fish

Healthy largemouth bass fingerlings were maintained at the aquaculture experimental facilities of the University of Arkansas Pine Bluff (UAPB). The study protocol was approved by the UAPB Institutional Animal Care and Use Committee (UAPB2021-03). Fish were acclimatized for two weeks in tanks with a capacity of 15 L and were fed with a 40% protein diet twice daily. Fingerlings with an average weight of 5 g were randomly assigned to five treatment groups each consisting of six replicates. Each replicate tank contained 15 fish. Water quality parameters were monitored using the Hach kit [28] and maintained within acceptable levels.

### 2.2. Edwardsiella Piscicida Isolates

*Edwardsiella piscicida* isolates (Table 1) were obtained from the Aquatic Research Diagnostic Laboratory (ARDL), Stoneville, MS, and confirmed as *E. piscicida* using species-specific multiplex PCR assays (Table 2). Heterologous *E. piscicida* isolates (S11-285, S17-335, S13-636, and S15-197) representing discrete phyletic groups (MLSA) [1,2,3,4]; Ref. [24] were used in the challenge study. The selected *E. piscicida* isolates were revived from cryostocks on TSA (Tryptic Soy Agar) plates supplemented with 5% defibrinated sheep blood and incubated for 24 h at 28 °C. Individual colonies from each of the bacterial isolates were expanded in 9 mL of Brain Heart Infusion (BHI) broth using a shaker incubator at 200 rpm (SHKE6000-7 ThermoScientific MaxQ, Marietta, OH, USA) for 24 h at 28 °C. Working cryostocks containing 20% glycerol were prepared and stored at −80 °C until use.

### 2.3. Fish Challenge

The pathogenicity of *E. piscicida* isolates originated from catfish belonging to different genetic clades was investigated in largemouth bass fingerlings. The LD_50_ (half minimal lethal dose) for each *E. piscicida* isolate was determined based on a 96 h time duration [31]. For the challenge, a 9 mL bacterial inoculum of each isolate prepared as mentioned above was expanded in 500 mL of BHI and incubated overnight at 28 °C in a shaker incubator at 250 rpm. Fish were not fed on the day of the challenge. Fish were challenged by immersion exposure to the bacterial isolates combined with mucus removal from the fish skin. Fish were anesthetized using MS-222 (30 mg/L), and mucus was removed from the fish skin using a bottle brush for 10 s. Estimated doses (Colony Forming Units (CFUs) per mL of water) of *E. piscicida* isolates were administered to fish by immersion exposure. Challenge doses were determined by standard plate counts for each isolate [32]. The immersion duration was 60 min, and the doses used were 9.2 × 10^9^, 9.7 × 10^9^, 4.1 × 10^9^, and 1 × 10^10^ CFU/mL for the S11-285, S17-335, S13-636, and S15-197 isolates, respectively (Table 1). The control group was exposed to BHI broth. The fish were monitored daily for any clinical signs of disease, and mortality was recorded for 15 days post-exposure. The water temperature was maintained at 25 ± 1 °C, and other water quality parameters were monitored throughout the study period to ensure that they remained within the acceptable levels. The percentage (%) of fish mortality was calculated using the following formula: percent mortality = (Fish mortality in the bacteria-exposed tank/Total number of fish in tank) × 100. To confirm the presence of *E. piscicida*, swabs taken aseptically from the posterior kidney of fish exhibiting clinical symptoms were cultured on TS (Tryptic Soy) blood agar plates.

### 2.4. Histopathology

Moribund fish from the infectivity trials involving all challenge isolates were used for histopathological analysis. Tissues from the infected fish were collected at time points ranging from 24 h to 10 days post-inoculation (dpi), and tissues from the uninfected group served as the controls. Moribund fish were euthanized using MS 222, and tissues (spleen, head kidney, and brain) were fixed in a 10% buffered formalin solution. The fixed tissues were decalcified (Cal-Ex, Fisher Scientific, Fair Lawn, NJ, USA), processed, and embedded in paraffin. The tissues were sectioned, stained with hematoxylin–eosin (HE), and examined using a BX-50 Olympus microscope (Olympus Optical, Tokyo, Japan). Representative images were captured with an Olympus DP72 camera and DP2-TWAIN-CellSens software (Olympus Optical, Tokyo, Japan).

### 2.5. Statistical Analysis

Fish mortality data were analyzed by a one-way ANOVA, with *p* ≤ 0.05 deemed statistically significant. Student’s *t*-test was employed to determine statistical differences among the treatment means. Mortality rates were presented as percentage (%) mortality ± SEM (standard error of the mean). Survival probability was estimated using Kaplan–Meier analysis.

## 3. Results

### 3.1. Clinical Symptoms of Largemouth Bass Infected with E. Piscicida

**Figure 1:**
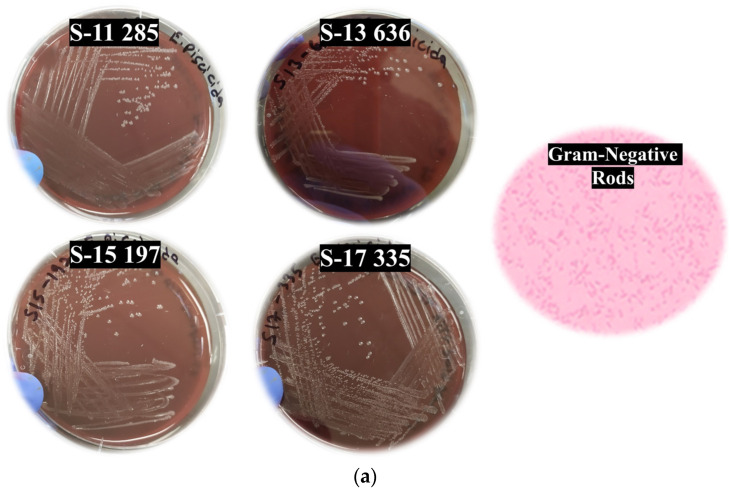

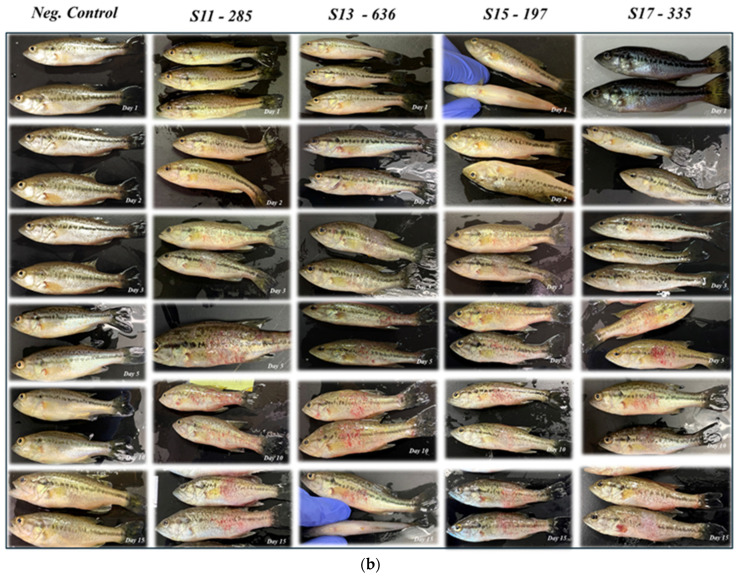
*Edwardsiella piscicida* isolates cultured on Tryptic Soy Agar with 5% sheep blood. (**a**) The colonies were white, punctate, and slightly hemolytic. (**b**) Gross pathological lesions observed in largemouth bass fingerlings experimentally infected with *E. piscicida* isolates. The infected fish exhibited external hemorrhages 120 h post-infection, while the control group did not exhibit any symptoms.

The behavioral symptoms of the infected fish included erratic swimming, slow reaction response, and inappetence. Externally, the scales were lost in some of the affected fish. The skin and muscle of the infected fish were ulcerated near the fins (Figure 1). Some of the diseased fish exhibited exophthalmia. Additionally, congestion and swelling were observed in the spleen and kidney of the diseased fish. Internal lesions were characteristic of bacterial infection with hemorrhages, necrosis, and inflammatory lesions. Swabs from the infected fish cultured on TSA blood plates yielded white, punctate, and slightly hemolytic colonies, confirming infection caused by *E. piscicida* (Figure 1).

### 3.2. Histopathology

**Figure 2:**
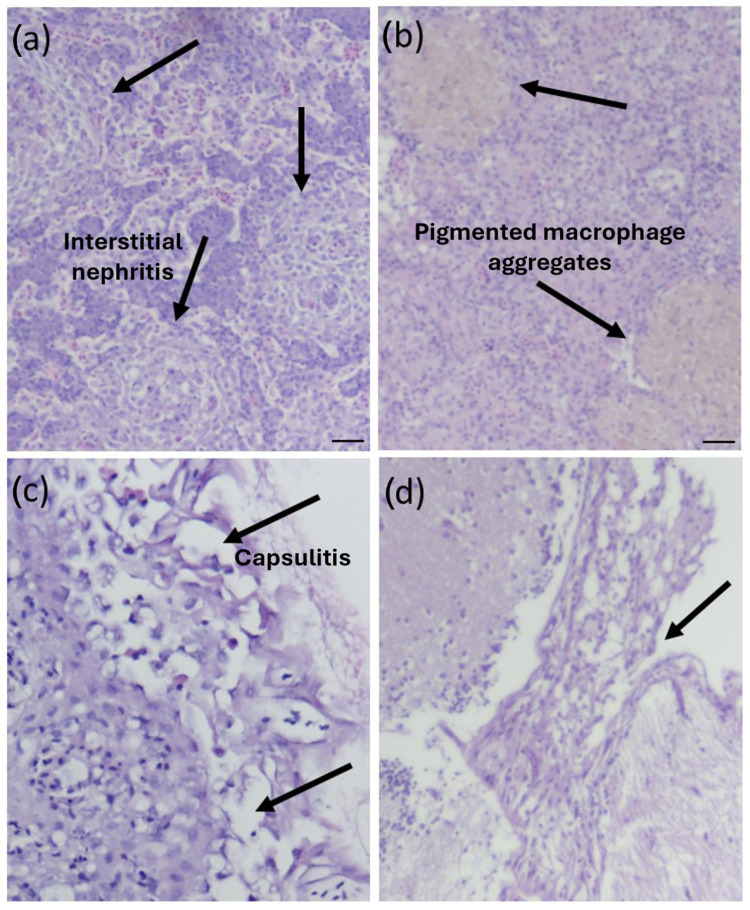
Histopathology analysis of the tissue sections (stained with hematoxylin–eosin) from largemouth bass infected with *E. piscicida*. The tissues indicated inflammatory lesions consistent with bacterial septicemia. (**a**) Anterior kidney with interstitial nephritis (black arrow), (**b**) spleen with splenitis and increased pigmented macrophage aggregates, (**c**) splenitis and capsulitis, and (**d**) brain tissues with meningitis were observed (scale bar = 20 μm).

Histopathological analysis of the tissue sections from largemouth bass infected with *E. piscicida* indicated lesions consistent with bacterial septicemia (Figure 2). However, the histology of the infected fishes did not reveal any specific trends for the tested *E. piscicida* genotypes (Figure 2). The anterior kidney of the infected fish (Figure 2a) presented interstitial nephritis accompanied by cell degeneration. Spleen sections of the infected fish had splenitis, increased pigmented macrophage aggregates, capsulitis, and were congested, with some cells displaying vacuolar degeneration (Figure 2b,c). The brain tissue sections from the infected fish showed meningitis (Figure 2d). Tissue sections from the control fish displayed normal texture and structurally intact cells.

### 3.3. Fish Mortality

**Figure 3:**
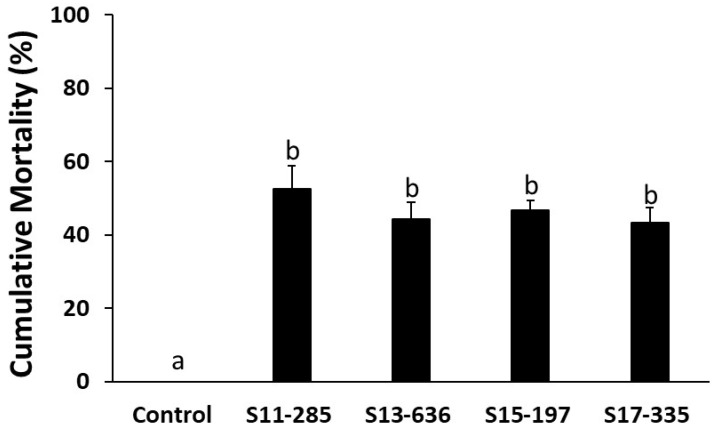
Percent mortality in largemouth bass fingerlings challenged with *E. piscicida* isolates (S11-285, S13-636, S15-197, and S17-335) belonging to different genetic clades [24]. Fish were challenged by immersion exposure (60 min) using the bacterial isolates combined with mucus removal from the skin. While there was significant mortality in fish exposed to the *E. piscicida* isolates compared to the control (a), the mortality caused by genetically distinct *E. piscicida* isolates (b) did not differ statistically.

**Figure 4:**
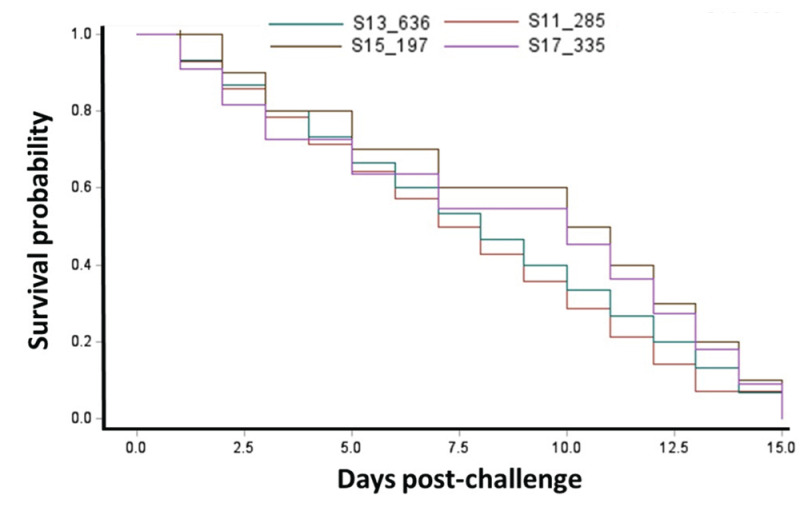
Kaplan–Meier survival analysis of largemouth bass (*Micropterus salmoides*) fingerlings subjected to immersion challenge with heterologous *E. piscicida* isolates from distinct genetic clades [24]. The Kaplan–Meier estimated 15-day survival rate (%) is shown. The control group exhibited no mortality throughout the duration of the experiment.

Largemouth bass fingerlings challenged with *E. piscicida* isolates (S11-285, S13-636, S15-197, and S17-335) indicated significant mortality compared to the control groups (Figure 3). The cumulative mortality in the experimentally infected largemouth bass ranged from 40 to 54% (Figure 3). There were no significant differences in mortality caused by the different genetic groups (differentiated by Multilocus Sequence Analysis (MLSA)) of *E. piscicida* in largemouth bass. Kaplan–Meier survival estimates (15-day survival rate) of largemouth bass fingerlings challenged with *E. piscicida* are provided (Figure 4).

## 4. Discussion

The genus *Edwardsiella* comprises five species, namely *E. piscicida*, *E. ictaluri*, *E. hoshinae*, *E. anguillarum*, and *E. tarda* [3,13,15,16]. *Edwardsiella piscicida* primarily affects fish, particularly those in aquaculture settings, and can cause severe losses due to its ability to infect a wide range of farmed, ornamental, baitfish, and sport fish species [3,4,5,6,7,8,9,10,11,14,15,16,17,18,19].

Our study indicated largemouth bass to be highly susceptible to catfish-originated *E. piscicida* isolates representing discrete phyletic groups [24], resulting in bacterial septicemia and associated fish death. The behavioral and external clinical abnormalities in the *E. piscicida*-infected largemouth bass included lethargy, loss of appetite, skin lesions, hemorrhages, and skin discoloration, as reported in previous studies [5,10,17,33,34]. Internally, the infected fish showed generalized septicemia, ascites, and inflammation in various tissues.

*Edwardsiella piscicida* infects susceptible fish through various routes, including ingestion, skin lesions, and gill surfaces, leading to systemic infection and mortalities. In most of the previous experimental infectivity studies, intracelomic (IC) or intraperitoneal (IP) injections of the host were employed. While these routes are reported to provide consistent infection results, the oral and immersion challenges with *E. piscicida*, *E. tarda*, and *E. anguillarum* do not reliably induce disease in cultured ictalurids [17]. However, the IC/ IP administration could circumvent innate host defense and limit natural infection pathogenesis and disease onset timing. In the current study, the experimental infection was achieved by immersion exposure combined with mucus removal from the fish skin, resulting in *E. piscicida* septicemia. Laboratory challenge models mimicking natural infection will be helpful in exploring the host–pathogen interactions, disease progression, and the pathogenicity of these bacteria.

Lesions caused by the genetically distinct *E. piscicida* isolates were typical of acute bacterial sepsis and cannot be easily distinguished grossly or histologically. Natural *E. tarda* or *E. piscicida* infections in fish reportedly progress from cutaneous hemorrhages to dermal ulceration [5,10,17,34,35,36]. While some petechial hemorrhages and surface discoloration were observed in some of the infected largemouth bass in this study, large ulcers were not recorded. The small size of the fish further limited the gross examination of the internal viscera. The fingerlings used in this study were much smaller than the stocker and market-size fish usually affected in commercial ponds. This could be the reason for the absence of the classic “hole-in-the-head” lesion often associated with chronic enteric septicemia of catfish [5,24,35] or *E. piscicida* septicemia.

Fogelson et al. (2016) reported *E. piscicida*-associated mortality in largemouth bass, as was observed in the current study [10]. Histopathology revealed multifocal areas of necrosis scattered throughout the heart, liver, anterior and posterior kidney, and spleen. Additionally, many of the necrotic foci were encapsulated or replaced by discrete granulomas and associated with colonies of Gram-negative bacteria [10]. The splenitis, nephritis, and encephalitis observed in the *E. piscicida*-infected largemouth bass tissues were consistent with the lesions caused by Gram-negative bacteria in whitefish (*Coregonus lavaretus*) [11] or *E. ictaluri* or *E. tarda* in channel catfish [37,38]. However, the observed lesions were different than those reported in the liver and kidney in the Japanese eel [39] and turbot (*Scophthalmus maximus*) [40] challenged with *E. tarda*. Striped bass (*Morone saxatilis*) infected with *E. tarda* had epithelial hyperplasia and necrosis in the lateral line canals and abscess formation in the anterior kidney and other internal organs [41]. It is likely that several of these previously reported *E. tarda* infections could be caused by *E. piscicida* [3]. Significant granulomatous lesions have been reported with *E. piscicida* or *E. tarda* challenges of largemouth bass [9] and barramundi (*Lates calcarifer*) [21], which were absent in *E. piscicida* challenges in the spotted sea bass, *Lateolabrax maculatus* [42], and in the current study. It is possible to have such lesions further in the course of the disease and with chronic infection.

Pathogenicity accounts for the strain’s ability to invade fish tissues, replicate within the host, and cause pathological changes indicative of disease [43]. Pathogenicity studies typically aim to elucidate various aspects of the bacterium’s interaction with the host, including its mechanisms of infection, virulence factors, host immune response, and disease progression. The catfish-derived *E. piscicida* isolates used in this study harbor virulence genes such as *hemolysin*, *adhesin*, *invasin*, etc., [24,25,26]. These can influence the bacterium’s ability to adhere to fish tissues, evade the host immune response, and produce toxins that contribute to tissue damage and disease progression.

The severity of infection can vary depending on factors such as the species of fish, environmental conditions, and the virulence of the particular strain of *E. piscicida*. Previous studies have reported the virulence of *E. piscicida* isolates in catfish, bass species, and tilapia. A study by [44] in Chinook salmon (*Oncorhynchus tshawytscha*) fingerlings, intracelomically challenged (~10^6^ CFU/fish) with 37 *E. piscicida* isolates belonging to six discrete MLSA clades, showed that peak mortality occurred 3–5 days post-challenge (dpc) regardless of isolates or genetic groups. The cumulative mortality ranged from 71 to 83%, suggesting an underlying genetic basis for strain virulence and potential host associations. The current study was unique as it looked at the susceptibility and host–pathogen dynamics of largemouth bass fingerlings and catfish-originated *E. piscicida* isolates representing discrete phyletic groups [24] and employed an immersion route of bacterial exposure. All of the tested *E. piscicida* isolates caused ~50% mortality in experimentally infected fish, indicating the high susceptibility of this economically relevant species. In addition, the mortality rate of largemouth bass fingerlings signifies that this life stage is also highly susceptible.

*Edwardsiella piscicida* has emerged as a devastating pathogen in the U.S. catfish aquaculture, responsible for significant mortality in food-size hybrid catfish, causing substantial economic loss [5,17]. With the intensification of aquaculture, the losses caused by *E. piscicida* septicemia have been increasing annually. By analyzing the farm-level production data and long-term disease trends in the U.S. catfish industry [22], Kumar et al. (2024) reported a negative economic impact of *E. ictaluri* and *E. piscicida*-associated septicemia ranging from −USD 15.5 to −USD 45.9 million/year.

Advancing our understanding of the pathogenicity of *E. piscicida* in a specific host facilitates the development of targeted management strategies, including vaccines, which is critical to industry profitability [45,46,47]. Studies by [7,34,47] have reported cross-protective immunity conferred by a live-attenuated *E. ictaluri* vaccine [48] against *E. piscicida* infection in channel and hybrid catfish, and this could be explored in largemouth bass.

By understanding the virulence of *E. piscicida* isolates in largemouth bass, management strategies can be tailored to target specific strains and minimize the impact of infections. Characterizing the *E. piscicida* virulence factors (such as adhesins, hemolysin, and secretion systems) contributing to septicemia in largemouth bass could be useful. Research on the host immune response (altered gene expression, immune cell populations, and cytokine production) associated with *E. piscicida* infection in largemouth bass is also desirable. Further studies on host–pathogen dynamics in food-size fish, the influence of environmental factors in disease progression, and variable challenge conditions are warranted.

## 5. Conclusions

Catfish-derived *E. piscicida* isolates used in the current study resulted in significant mortality in largemouth bass, indicating the pathogenicity of the heterologous isolates towards the host species. Though *E. piscicida* is reported to be highly pathogenic towards food-sized catfish, in this study, the bacteria caused heavy mortality in largemouth bass fingerlings, suggesting that this age group is also highly susceptible. Studies on the host–pathogen interaction using market-sized largemouth bass might provide additional insights. It might also be of interest to look at the effect of different environmental factors on the pathogenesis of *E. piscicida*. The results of this study extended our understanding of the pathogenicity of genetically discrete *E. piscicida* isolates in largemouth bass that could be used as a foundation for developing control strategies for *E. piscicida* septicemia.

## Figures and Tables

**Table 1:** Representative *Edwardsiella piscicida* isolates used in the infectivity trial with largemouth bass fingerlings. *Edwardsiella* isolates used in this study originated from farm-raised catfish in Mississippi, USA, and represented discrete phyletic groups [24]. The isolate ID indicates the location, year of isolation, and case number. Estimated doses (Colony Forming Units (CFUs) per mL of water) of heterologous *E. piscicida* isolates administered to fish by immersion exposure are provided.

Isolates	MLSA Clade	Estimated Dose (CFU/mL)
S11-285	MLSA 1	9.2 × 10^9^
S17-335	MLSA 2	9.7 × 10^9^
S13-636	MLSA 3	4.1 × 10^9^
S15-197	MLSA 4	1 × 10^10^

**Table 2:** Primers and probe used for the molecular confirmation of *Edwardsiella piscicida* isolates [4,16,29,30]. The species-specific PCR primers were developed based on *E. piscicida* genome sequences (Griffin et al., 2014 [4], GenBank accession no. JX866995, JX866998, JX866999, JX867000, JX867001, JX867002, JX867003) with the fimbrial subunit (130 bp) being the gene target. The probe, 6-carboxyfluorescein (6-FAM), was labeled with a fluorescent reporter dye on the 5′ end and quencher dye (black hole quencher 1) on the 3′ end.

Bacterial Strain	Primers	Sequence 5′–3′
*E. piscicida* (S11-285, S17-335, S13-636, and S15-197)	EP14529F	CTTTGATCATGGTTGCGGAA
	EP14659R	CGGCGTTTTCTTTTCTCG
	EP14615P	CCGACTCCGCGCAGATAACG

## Data Availability

The original contributions presented in this study are included in the article. Further inquiries can be directed to the corresponding author.
